# Supplementary material for: Parents Reaching Out to Parents: An Appreciative, Qualitative Evaluation of Stakeholder Experiences of the Parent Champions in the Community Project
Source: Children (Basel). 2022 Sep 27;9(10):1479. doi: 10.3390/children9101479 (PMC9600284; doi:10.3390/children9101479)
Supplement: Supplementary file 1 [file children-09-01479-s001.zip › Supplementary File S1 PCC Interview schedules-25th July 2022.pdf]

Interview questions

Parent Champion interviews

Timepoint 1

- What are the three most important things you learnt from your training?
- What worked well with the training you received and what could have been done better?
- What do you envisage will be/has been the most exciting aspect of your role?
- What do you think will be the biggest challenges you will face as a parent champion?
- What do you think your superpowers are?
- What are your memorable moments so far as a parent champion?

Timepoint 2

- What are your three best memories of being a parent champion?
- What challenges have you faced and how did you overcome these?
- What advice would you give to a new parent champion?
- Do you have any new superpowers?
- What would you like the 'government' to know about the project?

Parent interviews

- Did you find the visit from/contact with the parent champion helpful?
  - Prompts: why, what was helpful, was anything unhelpful, frightening?
- Were you able to ask questions about your baby/child?
  - Prompts: what sort of questions did you ask, who else could you have asked if you'd not spoken with a parent champion?
- Did you find it easy to understand the information the parent champion talked to you about?
  - Prompts: why was this so, how did they pitch the information?
- How much do you trust the information given by your parent champion?
  - Prompts: how much do you trust them compared to GP, nurse/HV, family member, other?
- Do you feel more confident about looking after your baby/child if they get poorly?
  - Prompts: why is this, what might you do differently now (e.g., when to see GP/hospital)?
- Would you recommend a parent champion to a friend?
- What's the most memorable thing that you learned from your parent champion?

Children's Centre Manager interviews

- What are your expectations of the project?
- Were your expectations achieved?
- What key elements of learning have you experienced in the project?
- What are your memorable moments?
- What extra does the Parent Champion bring to your Children's Centre?
- Is there anything that could be improved?

Children's Centre Group Leader interviews

- What 'extra' (memorable moments) did the engagement of the parent champion have on/within their activity/session?
- What worked well and what could have been done to enhance the involvement of the parent champion?
- What key learning/knowledge/skills were brought to the session that related to bronchiolitis?
- Do you think it's important to have Parent Champions in the Children's Centres and if so, why?

Core Team interviews

- What are your expectations of the project?
- Were your expectations achieved?
- What key elements of learning have you experienced in the project?
- What are your memorable moments?
