# Supplementary material for: Parents Reaching Out to Parents: An Appreciative, Qualitative Evaluation of Stakeholder Experiences of the Parent Champions in the Community Project
Source: Children (Basel). 2022 Sep 27;9(10):1479. doi: 10.3390/children9101479 (PMC9600284; doi:10.3390/children9101479)
Supplement: Supplementary file 1 [file children-09-01479-s001.zip › Supplementary File S2 PCC - Tables S1-S7-25th July 2022.pdf]

Table S1. Most memorable things from training

Factors	Exemplar quotes
Statistics and risk factors	• [Good to know the] facts for Liverpool… better than just national facts (PC8).<br>• Risk factors—deprivation in city (PC2).<br>• The highest levels in country are in Liverpool—air pollution and smoking and other risk factors (PC4).<br>• Learned about statistics in Liverpool and the number of children hospitalised (PC5).
Protection and prevention	• Learnt things to do, to tell and advise such as opening windows, the way you can tell a parent, if they are a smoker, that even one less cigarette a day can help (PC7).
Signs and symptoms	• Knowing that it's quite similar to a common cold but you need to look out for other things (PC1).<br>• Knowing the [breathing] sounds—the videos helped me to learn the noises (PC9).
How to engage with parents	• Compassion—don't blame people. Be careful about telling people off and blaming them (PC6).<br>• How to speak to parents who are at risk (low income), smokers, how to sit in on groups and approach parents (PC3).
Action to take	• Knowing when to take their child to GP, phone 111 or go to hospital—lots of parents very scared about treating child at home (PC1).
Other	• Opened my eyes to what the Children's Centres can do—thought they just did baby classes but…..if you have trouble they can help (PC7).<br>• Access to other resources like 'Care of the Chemist' (PC9).

Table S2. Delivery and accessibility information

Factors	Exemplar quotes
Accessibility of the information	• I related to her as she had children—I felt very comfortable (P20).<br>• She spoke on my level and spoke about her own children and experiences (P50)<br>• It wasn't a lecturey-type speech—she was having a chat so I didn't switch off—you tend to switch off if it's a lecture. She was friendly (P25).<br>• Helpful advice without scaring me—didn't frighten me—really valued this (P6).<br>• Helpful lovely and approachable—one of us—not bombarding us with information (P11).<br>• She didn't use any jargon; it was a conversation—advice and support—she came to me as a mum P58).<br>• Really informal, nice, chatty—not medical, not a lecture—she was friendly/easy going. She introduced the idea [in the group] and then spoke to us individually (P47).<br>• If I didn't understand, she broke it down more simple things. She did a good explanation about how it can develop and when to get more information (P45).
Videos and leaflets	• I always read over leaflets and digest things and then leave it to one side. But I've got it there parked if I need it to refresh myself (P26).<br>• She gave me three leaflets—me and my husband read them later—he didn't

	know about bronchiolitis (P28).<br>• She was showing the videos and the sounds—better than just talking and description. Having heard it, it is in your head (P16).<br>• Talked me through the symptoms and showed the video—I could see with own eyes on an actual child (P17).<br>• She had a little video of the tugging and head bobbing. The videos are really helpful—these help you not second guess (P11).<br>• The videos are really helpful—knowing what a normal sound is like and the weird noise (P9).<br>• The videos of how a sick child breathes and coughs helped me a lot. My child has never been ill, so before I would not have to compare whether the situation is serious or not (P18).<br>• The videos were upsetting to watch. She showed us kids 'suffering to breathe', a live example. It stayed with me, had a big effect. I could see it for what it is. It was horrible but I know what to look out for (P5).
Speaking a second language	• Definitely helpful. She's [cultural background] and I'm [cultural background]—cultural similarities. I didn't know that incense could aggravate a child's airway (P6).<br>• We had such a comfortable situation, we both use ['home' language], certainly helped me better understand the medical terms (P18).

Table S3. Parents learning about bronchiolitis

Factors	Exemplar quotes
Finding out about bronchiolitis	• It's common nearly all children get it. It can be severe, but not many children get it severely. Look for signs like the chest going in, lips going blue, struggling to breathe (P46).<br>• I hadn't had a clue about what she was talking about before. Didn't know how to deal with (P19).<br>• She explained that bronchiolitis is a virus which can affect breathing in young babies and in serious cases hospitalisation may be necessary (P36).<br>• She said it [bronchiolitis] could happen again and again and again. Now I'm prepared as I didn't know that before (P7).
Statistics and risk factors	• We struggled with my son with bronchiolitis a few times when he was a baby… I didn't realise it was so common—made me feel surprised (P58)<br>• She told me about the air pollution as well; that it's a problem (P15).<br>• She mentioned some statistics—it's so high in Liverpool!—I do take the children to lots of groups so they could catch it, I know (P34).
Protection and prevention	• She explained we can protect our children. It was helpful and straightforward information. She gave me tips to prevent it like wiping toys down and washing hands. Good to know I can protect him (P53).<br>• She told me about signs and that having candles around can be a harm to her chest and so can laundry powders—I can protect (P14).<br>• She told me in our culture we shouldn't use the nice smell (candles) too much—and to open windows and keep baby away from kitchen smells. I didn't know about this (P7).
Signs and symptoms	• I know about these serious signs now. I like knowing about worst case scenarios, this puts me at ease and although I'm very anxious, if I have information, it's better, I can be prepared (P45).<br>• Now I've got the information I need—the signs and whether it is more

	serious and what to do if I'm worried, if it's serious (P32).
Action to take	• Carry on taking steps—washing hands, cleaning and taking care and avoiding people who are coughing and sneezing. Good to hear this message reinforced (P26).<br>• Be alert. Don't be in a panic as it is probably absolutely fine. Be aware but don't turn a blind eye to little signs (P52).<br>• Good idea not to run straight to the hospital (P21).<br>• Call 111 if you need to or go to hospital if his tummy and breathing is going in and out like on the video (P7).<br>• I know not to rush straight to hospital. Stay calm and think (P57).
Outcomes	• It gave me confidence that if my child develops the disease, I will not miss any important symptoms (P18).<br>• good to be aware and I met up with a friend in the park and told her about it (P26).<br>• She told me about Care in the Chemist – that's really good (PC5).<br>• It's nice not to bother a GP with first mum nerves, I don't want to waste their time. I could clarify something with her [PC]- a 'middle man'—so you're not bothering doctor (P16).

Table S4. Reasons why parents trusted the information

Factors	Exemplar quotes
Trusting information from another parent	• It's better and easier to get information from a parent champion—harder to see and get information from a GP (P21).<br>• I've found that having a parent giving you information is better (than doctor or nurse)—they've got experience and they can draw on that—this makes it more real and useful (P25).<br>• Health professional information might be more scientific but information from [Parent Champion] was good and it was for the public. Good to have it presented like this (P48).<br>• She knows things like I do—it's real experience. Talking to someone like me [is better than talking to a GP]; I'm more able to understand things, she don't use big words (P52).
Trust engendered by association with Alder Hey	• I trust her completely; she also works with the NHS and it's NHS information (P14).<br>• I trust the information—she works at Alder Hey—they take looking after children's lives seriously (P26).<br>• It's too hard to get in touch with a GP, so it's good we have someone in the Children's Centre (P29).

Table S5. Enhancing confidence and decision-making

Factors	Exemplar quotes
Confidence informing decisions	• Before, I would have rung ambulance but now I'd go to the walk-in [centre] or go to GP (P54).<br>• Now we know the more serious signs we wouldn't rush to hospital if they weren't proper ill (P58).<br>• She did have a little cold after I'd seen [Parent Champion] so I watched the videos and checked if she had the 'raspy' noise and looked at the leaflets and checked with [Parent Champion]. She [baby] was fine—just a cold, but it did really help to talk (P25).<br>• Some of things I did when he had it when he was 2 weeks old, I'd do a bit different. I went straight to the hospital and they said it was viral and they couldn't do much and sent me home. He was OK. I know what to do now. (P29).<br>• I've called the Children's Centre a few times about other things, I'd never think about calling the Health Visitor if he was sick. I've only heard from them once since he was born (P46).<br>• My baby had been suffering from a cold the last week and I did take her to the GP to get checked but I was confident it wasn't RSV as I remember some of the signs [Parent Champion] told me to look out for (P50).

Table S6. Parent Champions' superpowers

Factors	Exemplar quotes
Superpowers	• I've learned about myself**.** I'm quite nervous and shy but people don't seem to notice—so that's a superpower! (PC1)<br>• Being able to improvise, change and adapt to different people: some people want more formal, some want more chatty (PC2).<br>• Making parents feel I'm never judging. When I had my little girl, I felt judged by everyone. I can get on their same wavelength. (PC3).<br>• 100% multitasking—I don't even think about it now—it's helped to juggle things at home as well (PC4).<br>• Speaking two languages and being able to speak English in a way they understand. I can feel for parents. (PC5)<br>• The world is unfair and unkind, but I can make them laugh and have fun (even though bronchiolitis is serious)' (PC6)<br>• I've got a positive attitude and a lot of energy, I think maybe they feel my energy (PC7).<br>• I share my smile and my positive thoughts and feelings and it's mum and making connections (PC8).<br>• Being a mum—we often underestimate ourselves! (PC9).

Table S7. Advice for a new Parent Champion

Factors	Exemplar quotes
Advice for a new Parent Champion	• Ease into it take your time, get to know the parents, get yourself in the centre—the staff there are key, they know where everything is (P4).<br>• Be prepared to get involved in a wide range of activities not just bronchiolitis and help in the Centre… It's like a circle—I help centre, centre helps me (P2).<br>• Get to know parents and chat to them – I find out about their babies so I can make what I say personal (P6).<br>• Don't be nervous—you don't need to know everything—you don't need to be a GP (PC1)<br>• Believe in yourself (P9).<br>• Enjoy it, you'll be happy with the inner of your heart (PC5).<br>• Keep it simple—don't use complicated words like dehydration people don't always know what it means, people get frightened and think the information is too hard (P7).
